# Retail chain pharmacy opioid dispensing practices from 1997 to 2020: A content analysis of internal industry documents

**DOI:** 10.1016/j.dadr.2023.100199

**Published:** 2023-11-02

**Authors:** Clever Chiu, Anthony Wong, James Chhen, Alfred-John (A.J.) Roderos, Dorie E. Apollonio

**Affiliations:** School of Pharmacy, University of California San Francisco, San Francisco, California, USA

**Keywords:** Pharmacies, Analgesics, Opioid, Prescriptions, Policy

## Abstract

**Context:**

Lawsuits have determined that community pharmacy chains played a crucial role in the opioid epidemic. However, little research has assessed community pharmacy practices. This study sought to understand the contribution of pharmacies to the opioid epidemic through improper opioid prescription dispensing.

**Methods:**

We conducted an observational, retrospective content analysis that assessed the opioid dispensing practices of a retail community pharmacy chain, Walgreens, using pharmaceutical industry documents released in litigation between 1997 and 2020. The documents were retrieved from the Opioids Industry Document Archive (OIDA) at the University of California, San Francisco and reviewed to identify themes and identify organizational practices.

**Findings:**

We identified four primary factors that may have contributed to improper opioid dispensing practices: store-level procedures, management pressure, distribution center activities, and pharmaceutical company sponsorship. Stores dispensed opioid prescriptions without resolving red flags, management pressured pharmacists to fill more opioid prescriptions, distribution centers failed to investigate high volume orders, and pharmaceutical companies sponsored pharmacist continuing education advocating for opioid pain management.

**Conclusions:**

Our findings suggest that Walgreens may have contributed to the early prescription opioid epidemic through improper opioid dispensing and also identify key practices that could be reformed to reduce the risk of future inappropriate dispensing of addictive and potentially harmful medications.

## Highlights

**What was already known?**

• Multiple lawsuits have determined that community pharmacy chains played a crucial role in the opioid epidemic, which led to over 500,000 opioid overdose deaths between 1997 and 2020.
• Past research has documented how providers were encouraged to increase opioid prescribing, but much less is known about the role of community pharmacies.

**What this study adds?**

• We identified four primary factors that contributed to improper opioid dispensing practices: store-level procedures, management pressure, distribution center activities, and pharmaceutical company sponsorship.
• Our findings suggest that Walgreens contributed to the early prescription opioid epidemic through improper opioid dispensing.
• We identified key practices that could be reformed to reduce the risk of future inappropriate dispensing of addictive and potentially harmful medications.

## 1 Introduction

Prescription opioids are a powerful class of medications used to treat moderate to severe pain (Rosenblum et al., 2008). In the United States, they have been essential in treatment following surgery, traumatic injury, and cancer (Rosenblum et al., 2008). Although natural opioids (opiates) such as morphine have existed for thousands of years, prescription opioid use increased substantially during the 1990s when addressing pain became a higher priority for providers (Bandyopadhyay, 2019). Opioid-related overdoses and deaths soared, leading to a public health crisis termed the opioid epidemic. Between 1999 and 2020, 564,000 people died from opioid overdoses and prescription opioids accounted for 263,000 of those deaths (CDC, 2023; CDC, 2022a). Between 2006 and 2012, prescription opioid dispensing increased to more than 255 million, an estimated 81.3 prescriptions for every 100 persons (CDC, 2022b). The opioid epidemic has been characterized by three waves. The first wave began in the 1990s with increased opioid prescribing, the second in 2010 with an increase in heroin use after stricter prescription drug monitoring programs were implemented, and the third in 2013 with increased use of both prescription and illicit synthetic opioids such as fentanyl (Bedene et al., 2022). The majority of people using illicit opioids in the second and third waves of the opioid epidemic developed opioid use disorder as a result of prescription opioid use (Van Zee, 2009).

The opioid epidemic was fueled by a series of events, including the marketing of a new opioid formulation, OxyContin, in the mid-1990s (Bedene et al., 2022; Van Zee, 2009; Cicero et al., 2005). A 1980 letter had suggested that opioids were not addictive, stating that “despite widespread use of narcotic drugs in hospitals, the development of addiction is rare in medical patients with no history of addiction” (Porter and Jick, 1980, p. 1). This letter was cited over 600 times as evidence of low opioid addiction risk for pain management (Leung et al., 2017). It was later revealed that this letter was funded by pharmaceutical companies (Gale, 2016). In response, editors at the New England Journal of Medicine, which had published the original letters, added a note in 2017 that stated, “For reasons of public health, readers should be aware that this letter has been ‘heavily and uncritically cited’ as evidence that addiction is rare with opioid therapy.” Beginning in the 2000s, multiple lawsuits were filed by state and local governments against pharmaceutical manufacturers for costs associated with the opioid epidemic, resulting in settlements with companies including Purdue, Cardinal, and McKesson pertaining to improper marketing and failure to act on suspicious orders (Meier, 2007).

Past research has documented how providers were encouraged to increase opioid prescribing, but much less is known about the role of community pharmacies. Prescription opioids are purchased from pharmacies, and proper dispensing is essential in preventing harm (Bedene et al., 2022). One study of testimony submitted in a lawsuit against CVS in West Virginia found that the company failed to implement suspicious order monitoring (SOM) due to a corporate attitude that relied only on workers to detect suspicious orders rather than having effective controls in place (Gonick, 2022). Studying the practices of retail chain pharmacies has historically been difficult given that there is limited information available on internal decision-making within organizations (Bero, 2003). Recent research on the opioid epidemic has addressed this issue by assessing internal documents released in litigation (Yakubi et al., 2022; Caleb Alexander et al., 2022), consistent with established practices pioneered in extensive academic research on the tobacco and food industries that have led to changes in policy and clinical practice that protect public health (Bero, 2003; Gunnarsson et al., 2023; Zaltz et al., 2022; Steele et al., 2022; Carriedo et al., 2022). In this research, we sought to identify potential practices of a retail community pharmacy that affected dispensing, understand their potential contribution to the opioid epidemic, and determine whether their practices could be modified to minimize future risks.

## 2 Methods

We conducted an observational, retrospective content analysis that assessed the opioid dispensing practices of a retail community pharmacy chain, Walgreens, using pharmaceutical industry documents released in litigation between 1997 and 2020.

The documents were retrieved from the Opioids Industry Document Archive (OIDA) at the University of California, San Francisco, in collaboration with John Hopkins University. As of September 28, 2023, OIDA held more than 3.1 million documents, containing more than 12.6 million pages that were made public during lawsuits against pharmaceutical companies, distributors, and pharmacies (OIDA, 2023). These documents include correspondence, presentations, spreadsheets on marketing budgets and sales performance, hotline complaints, detailed business plans, DEA investigations, trial transcripts, records of sales contacts and invoices, and details on the development and presentation of continuing education modules, among others.

Using the key term “Walgreen”, we reviewed three collections in the archive pertaining to Walgreens community pharmacies: Florida Walgreens Litigation Documents, Ohio Pharmacy Litigation Documents, and San Francisco Walgreens Litigation Documents. However, the Ohio Pharmacy Litigation Documents collection is not specific to Walgreens and includes other major community pharmacy defendants like CVS and Walmart pharmacies. Collectively, these three collections contained 630 documents, excluding duplicates, and a total of 46,345 pages. All documents from these three collections were reviewed by four authors ([redacted]) in the second year of PharmD training, each of whom had at least 208 hours of practice experience in community practices. Each document was read, documented in a spreadsheet along with key points and summaries, and reviewed for relevance. The first 20 documents were jointly coded by this team in consultation with a UCSF librarian responsible for the archive and a fifth author ([redacted]) with over 20 years of industry documents research experience. Following this initial review and calibration, the team reviewed the remaining documents in the collections. At least 2 of the 4 lead authors reviewed each document before selecting it for inclusion, exclusion, or for further review. Documents marked as in need of further review were assessed by the entire study team. We excluded documents in which opioid data could not be differentiated from non-opioid data. Research was conducted between January and April 2023.

Our analysis relied on modified grounded theory, in combination with description of the data to identify themes within the documents and assess associations between organizational practices and opioid dispensing. Modified grounded theory consists of data collection, data categorization, theme classification, and tying categories and themes to one core idea (Alnsour, 2022). We conducted sub-analyses by locations of Walgreens pharmacies identified in each collection: Florida, Ohio, and San Francisco.

## 3 Results

**Fig. 1:**
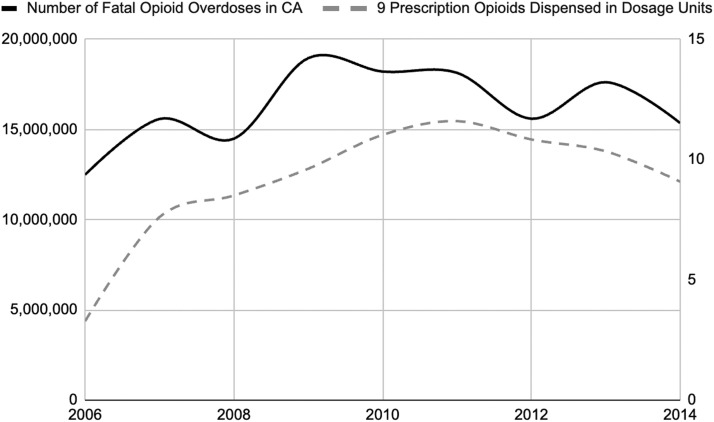
Trends in fatal opioid overdoses compared to the number of opioids dispensed in San Francisco, CA, 2006–2014. As Walgreens in San Francisco increased their opioid dispensing represented by dosage units dispensed (indicated by the left axis and gray dashed line), there was a corresponding increase in the number of fatal opioid overdoses in San Francisco (indicated by the right axis and black solid line). The nine prescription opioids indicated here include codeine, fentanyl, hydrocodone, hydromorphone, methadone, morphine, oxycodone, oxymorphone, and tapentadol. After 2011, there was a decrease in opioid dosage units dispensed, however fatal opioid overdoses continued to increase after 2012 due to heroin and synthetic opioids becoming more prevalent. Source: UCSF Opioid Industry Documents Archive and Center on Substance Use and Health of San Francisco.

Of the 630 documents in the three collections, we excluded one document containing non-opioid and opioid data that could not be analyzed separately. Of the remaining 629 documents, 21 documents were directly relevant to the research question, consisting of corporate correspondence, DEA orders to show cause, and opioid dispensing data. Walgreens pharmacies were dispensing abnormally large amounts of opioids from 2006 to 2014, during the peak of the prescription opioid epidemic and corresponding with the upward trend in fatal opioid overdoses; Fig. 1 shows this association in San Francisco (Walgreens Distribution and Dispensing of Nine Prescription Opioids in Dosage Units by Walgreens Pharmacy, by Year : For 2006-2020, in San Francisco, California, 2020; Coffin et al., 2022). Data for Walgreens pharmacies in Florida and Ohio was not included in the archives at the time of research.

**Fig. 2:**
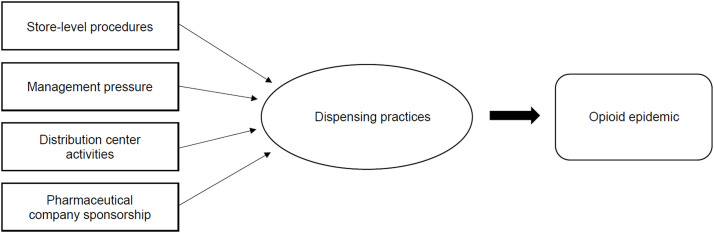
Four factors identified as contributing to improper opioid dispensing practices. Store-level procedures, management pressure, distribution center activities, and pharmaceutical company sponsored influenced opioid dispensing practices. Source: generated by the authors.

After reviewing relevant documents, we categorized our findings into four main factors that contributed to improper opioid dispensing practices: store-level procedures (discussed in 3 documents), management pressure (10 documents), distribution center activities (5 documents), and pharmaceutical company sponsorship (4 documents) (Fig. 2). Two of the documents we identified contained information relevant to multiple factors, and one document was relevant to all of the results.

### 3.1 Store-level procedures

Different Walgreens locations had varying store-level procedures associated with discrepancies in opioid dispensing practices. A recurrent theme in stores that filled more prescriptions was dispensing opioids with unresolved red flags, defined as indications that prescriptions needed further investigation before dispensing (Table 1) (US DEA, 2012b). These red flags included: (a) individuals who presented recurring prescriptions from the same prescribers for either the same drug or drug combinations in identical quantities, (b) individuals who presented prescriptions issued by multiple prescribers or providers who lacked authority to prescribe controlled substances, (c) individuals who shared similar addresses or resided in locations that were distant from either the pharmacy or prescriber, (d) individuals who paid for controlled substances in cash and/or with non-insurance discount cards, and (e) early refills for repeat customers (US DEA, 2012b). In these cases, oxycodone was the most commonly prescribed opioid, and was often dispensed in combination with other controlled substances that had high potential for abuse, known as opioid cocktails (US DEA, 2012b).

**Table 1:** Summary of findings from DEA investigations into Florida Walgreens stores, 2012-2013.

DEA ORDER TO SHOW CAUSE AND IMMEDIATE SUSPENSION OF REGISTRATION	
Walgreens Pharmacy Store Number & Address	Key Red Flags
#03099: 1525 Colonial Blvd, Fort Myers, FL 33907	230 dosage units of oxycodone 30 mg in combination with alprazolam and carisoprodol issued by Hill MD were dispensed early by #03099 to a customer on several occasions. Identical prescriptions for 60 dosage units of oxycodone 15 mg and 120 dosage units of oxycodone 30 mg issued by Posca MD were repeatedly filled by #03099.
#03629: 12028 Majestic Blvd, Hudson, FL 34667	354 controlled prescription drugs issued by Abaunza MD were filled by #03629. Abaunza's medical office in Miami is over 300 miles away from Hudson, FL. Prescription combinations of oxycodone 30 mg, hydromorphone 8 mg, and alprazolam 2 mg issued by Dr. Abaunza were repeatedly dispensed by #03629 to a customer who paid in cash.
#03836: 9332 US Hwy 19, Port Richey, FL 34668	Prescription combinations of oxycodone, hydrocodone, and alprazolam issued by Abaunza MD of Miami, FL were repeatedly filled by #03836 to customers who lived in various states – AL, KY, OH, and TN. These customers traveled a minimum of 1,500 miles round trip to obtain these prescriptions.
#04391: 2501 Virginia Ave, Fort Pierce, FL 34981	At least 40 prescriptions for hydrocodone, and oxycodone 30, 60, and 80 mg for a returning customer were filled by #04391 who obtained these opioid prescriptions from ten different physicians who were all located approximately 144 miles away from #04391. At least seven prescriptions were filled by #04391, despite warnings printed on prescription labels beneath various doctor's names and recorded in the pharmacy's electronic dispensing log. Prescriptions combinations of oxycodone and alprazolam issued by Harman DO were repeatedly dispensed ten days early by #04391, in which these prescriptions included 210 dosage units of oxycodone.
#04727: 4950 S US Hwy 1, Fort Pierce, FL 34952	Nearly identical prescriptions for oxycodone 15 and 30 mg and alprazolam 2 mg issued by Carrozzella MD were dispensed within a 10-minute period to five customers who paid with non-insurance discount cards. Carrozzella's medical office in Kissimmee is 138 miles away from Fort Pierce, FL. Prescription combinations of oxycodone 15 and 30 mg and alprazolam 2 mg issued by Dr. Carrozzella were dispensed to three customers who provided new Florida identification cards indicating the same place of residence.
#06997: 785 Lockwood Blvd, Oviedo, FL 32765	At least 64 prescriptions for oxycodone 30 mg issued by Wicks MD were filled by #06997, despite his expired, out-of-state DEA registration. Dr. Wicks’ office was based in Visalia, CA. More than 27,000 dosage units of oxycodone 30 mg issued by Dr. Wicks were dispensed by #06997. Oviedo Police Chief notified both Walgreens Corporate Headquarters and #06997 of several arrests of regular customers for the illegal distribution of oxycodone. Despite being notified by local law enforcement, #06997 continued to fill oxycodone prescriptions to these customers.

The DEA found that six Walgreens locations consistently failed to exercise their responsibility to ensure that controlled substances were dispensed pursuant to prescriptions issued for legitimate medical purposes by practitioners acting within the usual course of their professional practice. Source: UCSF Opioid Industry Documents Archive; data compiled by the authors.

In San Francisco specifically, there was evidence that Walgreens stores dispensed opioid prescriptions that lacked prescriber DEA numbers, which are legally required. Between 2005 and 2020, nearly 20 % of all opioid prescriptions dispensed at these stores lacked a prescriber DEA number or a unique prescriber DEA number and over 97 % of all opioid prescriptions lacked a diagnosis code (see Supplement); these rates were higher than in other stores outside San Francisco (see Supplement)(Summary of Walgreens Dispensing Data Quality, from 2005-2020 : For Walgreens Stores in San Francisco, Walgreens Stores Outside San Francisco, and 12 Selected Walgreens Stores in San Francisco, 2020).

Additionally, emails sent within the company in 1997 indicated that some stores sought to increase narcotics inventory as much as 8-fold in 24-hour stores, informed high prescribers in the area that those stores always had an adequate inventory, and allowed select stores to accept call-in orders for CII drugs (controlled substance schedule II, meaning drugs that have acceptable medical uses but also a high potential for abuse and dependence) (Gasdia, 1997). This strategy was referenced positively on the grounds that “doctors will have the assurance that the pain meds will be filled by a pharmacist less likely to question his/her prescribing habits” (Gasdia, 1997, p. 3).

### 3.2 Management pressure

The documents also suggest that pressure applied by Walgreens management may have also contributed to improper opioid dispensing on both the store and corporate level (Walgreens (Firm), Apis (Firm), 2017; Raval, 2010; Jaeger, 2018; Walgreens (Firm), Apis (Firm), 2013b, Apis (Firm), 2013a, Apis (Firm), 2015).

**Fig. 3:**
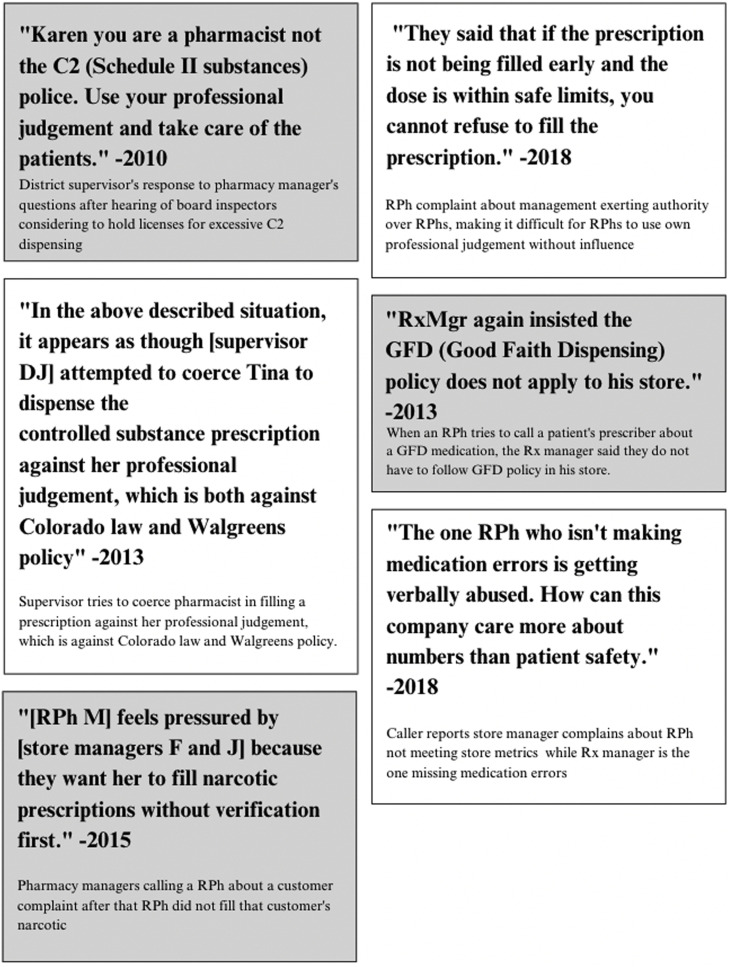
Compilation of reported incidents and management responses related to opioid dispensing. These are quotes and context from a selection of multiple incident reports submitted by individual employees to the Walgreens employee hotline that involved conflicts between management and individual RPhs regarding when to fill opioid prescriptions under the Good Faith Dispensing policy which guided Walgreens in dispensing controlled substances. Source: UCSF Opioid Industry Documents Archive.

Internal investigation forms from the Walgreens employee hotline from 2010 to 2018 revealed multiple incident reports at the store level that detailed disagreements between registered pharmacists (RPhs) and district and store managers on what constituted appropriate opioid dispensing (Fig. 3) (Walgreens (Firm), Apis (Firm), 2017; Raval, 2010; Jaeger, 2018; Walgreens (Firm), Apis (Firm), 2013b, Apis (Firm), 2013a, Apis (Firm), 2015). The most common theme in these complaints related to when to dispense a narcotic prescription. These included incidents in which managers indicated to pharmacists that the prescriptions they did not fill should have been filled, particularly after receiving customer complaints. Managers applied different criteria on when not to fill a prescription, creating concerns among pharmacists that they could not practice using their professional judgment while under constant surveillance by management. One pharmacist who was concerned about Board of Pharmacy inspectors holding licenses asked their district supervisor how to approach narcotic dispensing, and the supervisor responded, “Karen you are a pharmacist not the C2 [Schedule II] police. Use your professional judgment and take care of the patients” (Raval, 2010, p. 1). Pharmacists expressed concerns in incident reports that decisions to fill specific prescriptions were imposed by management, who viewed refusal as poor customer service and did not defer to pharmacists’ clinical judgment, leading to improper dispensing (Walgreens (Firm), Apis (Firm), 2017; Walgreens Code of Business Conduct Lead: IC10001237824, 2014; Gubbins, 2010; Walgreen Co., 2010; Bratton, 2013).

Incident reports completed by pharmacists also detailed concerns about metric and bonus structures that could lead to improper dispensing. These included metrics such as having a prescription ready to be sold by a certain time. One report filed in Orlando, FL in August 2014 related to a pharmacist being pressured by their manager to fill more opioid prescriptions, which was perceived to be related to managers receiving higher compensation when the store filled more prescriptions (Walgreens Code of Business Conduct Lead: IC10001237824 ,2014). In another report filed in Chicago, IL in July 2017, a pharmacist was criticized for having longer times to prepare a prescription to be filled, even though that pharmacist was the one identifying the most errors; at the same time, the pharmacy manager was dispensing prescriptions with errors (Walgreens (Firm), Apis (Firm), 2017).

At the corporate level, Walgreens management expressed concerns about stores with lower levels of opioid dispensing. A 2010 email from the Walgreens corporate office noted that certain Florida stores were filling lower amounts of oxycodone prescriptions and proposed investigating these stores, asking, “Are we turning away good customers?” (Gubbins, 2010, p. 1). As noted in incident reports described above, the Walgreens corporate office had created an incentive program to increase compensation for pharmacy managers in stores that filled more prescriptions. A 2010 document outlined how the bonuses were calculated, showing a formula based on 12-month prescription sales (Walgreen Co., 2010). This created another point of influence that could lead to improper dispensing, and in 2013, it led to a DEA-Walgreens agreement in which Walgreens agreed to exclude controlled substance prescriptions from calculations of compensation (Bratton, 2013).

Ultimately, both the internal investigation forms and corporate documents suggest that pressure from management at the corporate and store levels was directed onto individual RPhs in an effort to influence their professional judgment and lead to improper dispensing (Walgreens (Firm), Apis (Firm), 2017; Walgreens Code of Business Conduct Lead: IC10001237824, 2014; Gubbins, 2010; Walgreen Co., 2010; Bratton, 2013).

### 3.3 Distribution center activities

The archive also included evidence suggesting that three Walgreens’ distribution centers—Jupiter in Florida, Perrysburg in Ohio, and Woodland in California—that supplied medications to Walgreens retail pharmacies in their respective regions may have contributed to the improper opioid dispensing. We focused specifically on the Jupiter distribution center, where opioid distribution practices were most extensively documented. There was limited data on distribution centers in Ohio and California and no documentation of whether DEA registration was suspended at these two locations in the archive. Audits and investigations showed that the Jupiter distribution center failed to implement needed improvements in monitoring and processing of suspicious orders, as well as additional policies and procedures regarding the ordering, processing, and reporting of Schedule II drugs (Walgreens (Firm), 2008).

The Jupiter distribution center shipped abnormally high amounts of oxycodone to Walgreens retail pharmacies in Florida (US DEA, 2012a). As of 2009, Jupiter was the largest distributor of oxycodone products in Florida, supplying 3 of the top 100 oxycodone purchasers. Within three years, that number rose to 44 and Jupiter was deemed to be an “imminent danger to the public health and safety;” as a result, its DEA registration was suspended, which prohibited the facility from continuing to distribute controlled substances (US DEA, 2012a, p. 1).

Between November 2010 and March 2011, the Police Chief of Oviedo, Florida, wrote 16 letters to Walgreens pharmacists and corporate executives, including the President and CEO, detailing over 120 arrests made regarding the diversion and illicit use of oxycodone (US DEA, 2012b). The majority of the arrests were made at two Oviedo Walgreens pharmacies (Chudnow, 2010). Despite these repeated letters and reports, distribution data shows that Walgreens and Jupiter repeatedly distributed high quantity orders to its retail pharmacies. Specifically, one Oviedo (store #06997) received monthly dosage orders of oxycodone 30 mg that increased from 75,300 to 145,300 dosage units monthly over the span of five months (US DEA 2012a). In 2011, the average retail pharmacy in the US was purchasing approximately 73,000 dosage units of oxycodone, while Jupiter was distributing between 1.19 million to 2.21 million dosage units of oxycodone to six of its retail pharmacies in Florida (see Supplement) (US DEA, 2012a). At least 43 Walgreens pharmacies in Florida purchased over 500,000 dosage units in that year (US DEA, 2012a).

In January 2011, internal emails between the manager of Rx Inventory drug stores and the Schedule II function manager at Jupiter detailed a conversation expressing concern about the validity of orders from a series of stores, including store #03836 in Port Richey, FL, which had ordered an average of 362 bottles per week of oxycodone 30 mg in the previous 13 weeks (Martin, 2011). Nonetheless, purchasing data reveals that store #03836 still received 1406,000 dosage units of oxycodone in 2011 (US DEA, 2012a). In August 2011, Walgreens Loss Prevention tried to enact an order limit of 100 bottles of oxycodone 30 mg for the Hudson Walgreens store (US DEA, 2012a). However, the limit was not enforced; between September to December 2011, Jupiter shipped five orders consisting of 113 to 331 bottles apiece to that location (US DEA, 2012a).

### 3.4 Pharmaceutical company sponsorship

Purdue partnered with Walgreens and sponsored Walgreens’ continuing education (CE) programs for its pharmacists from 1997 through 2001. Medical liaisons from Purdue trained pharmacists attending these CE programs on pain management topics such as “pain management for pharmacists,” “managing pain in the elderly,” and “should I dispense this?” (Seid, 2001, p. 2). In an email exchange between Purdue and Walgreens corporate leaders in 2001, a Purdue representative mentioned including “patient testimonials at these programs to help the pharmacist in understanding the need for high dosage levels” of opioids (Sposato, 2001, p. 2). In another email, a Purdue corporate leader stated “in exchange for sponsoring this event, Purdue gets exclusive promotion rights and what I'd call ‘guaranteed’ stocking of future products at specific key stores” (Denning, 2000, p. 1). Purdue corporate leaders also reached out to Walgreens stores by having their sales representatives “forge strong partnerships with his chain pharmacies. This is one way to deal with the problem of pharmacists wanting to substitute MS Contin prescriptions” (Gasdia, 1997, p. 1). Purdue corporate leaders stated in an email that “there is so much misinformation at the retail pharmacist level and the last thing we need is a retail pharmacist refusing to fill or questioning a prescription that one of your reps worked so hard to generate” (Scifo, 2001, p. 3).

## 4 Discussion

Our study identified four factors that contributed to inappropriate dispensing of opioids and likely worsened the opioid epidemic: a combination of store-level procedures, management pressure, distribution center activities, and pharmaceutical company sponsorship. Although most of the data we reviewed was specific to Walgreens community pharmacists, the pressure to dispense opioids inappropriately often came from external sources. At the store-level, the pressure to meet certain metrics established by management likely contributed to red flags being left unresolved and prescriptions filled. Metrics assessing the number of prescriptions filled are important in assessing the performance of a store and identifying ways it can improve over time. However, including addictive medications such as opioids in these metrics creates perverse incentives to elevate profits over patient safety, given that these medications are high-risk for dependence, abuse, and addiction. Eliminating the demand to dispense higher numbers of opioid prescriptions allows stores and individual pharmacists to investigate red flag opioid prescriptions and ensure patient safety, and regulators should consider policies that require store metrics to exclude addictive medications from metrics assessing numbers of prescriptions filled. At the management level, individual pharmacists need legal protection that allows them to use their clinical judgment without store managers attempting to influence outcomes. We identified multiple employee hotline complaints that detailed the extent to which some store managers emphasized filling more opioid prescriptions over patient safety. Filling prescriptions for controlled substances involves a multi-step verification process and as a result, requires more time than filling prescriptions for other medications. This store management pressure may have been generated by low staffing levels or other time and resource constraints; if so, setting and enforcing minimum staffing levels might help prevent inappropriate dispensing of addictive medications in the future.

At the distribution center level, specifically in Florida, there were inadequate monitoring systems in place to detect and deter suspicious orders, which was discovered by the DEA in 2012 after it had led to some individual store locations receiving the same quantities of oxycodone in a month than most stores received in a year. After the suspension of Jupiter's distribution center license in 2012 and the imposition of large fines, the DEA investigated additional distribution centers and issued more fines for similar concerns (USAO, 2015). Further research is needed to determine how to prevent distribution centers from accepting suspicious orders.

Pharmaceutical company involvement in continuing education programs was driven by a clear profit motive in the case of Purdue's efforts to reach Walgreens’ pharmacists. Purdue sought to influence Walgreens pharmacists to become comfortable dispensing higher dosage opioids. Purdue's focus on generating profit by selling a high volume of OxyContin was apparent in an email exchange that stated that Walgreens would guarantee stocking of Purdue's future products as a quid pro quo for Purdue's sponsorship. These agreements between Purdue and Walgreens were intended to lead to increased opioid dispensing and may have helped fuel the opioid epidemic. Organizations such as “No Free Lunch” have argued that health care providers should not accept resources from pharmaceutical companies because it generates a conflict of interest; at a minimum, pharmacists and other providers should have the right to opt out of continuing education created by pharmaceutical companies even if it is sponsored by their employer (Abbasi, 2003).

This research on the practices of a retail community pharmacy provides critical guidance needed to assess current pharmacy practices and develop guidelines for dispensing that reduce opioid misuse and overdoses, as well as reduce the risk of improper dispensing for other medications with the potential for misuse and harm. Taken as a whole, our findings suggest patterns and trends in organizational practices at Walgreens that encouraged inappropriate opioid dispensing. Despite concerns expressed by registered pharmacists and law enforcement, Walgreens management did not change these opioid dispensing practices in response to increases in opioid overdoses until 2011. Around 2011 to 2012, Walgreens addressed this issue by updating and reinforcing its Good Faith Dispensing (GFD) policy, which outlines required verification steps prior to dispensing any medications, with a greater emphasis on opioids and each pharmacy team member's role in the process (Mail, 2012). However, our findings suggest that some problematic practices persisted until at least 2020 (Walgreens Distribution and Dispensing of Nine Prescription Opioids in Dosage Units by Walgreens Pharmacy, by Year : For 2006-2020, in San Francisco, California, 2020; Coffin et al., 2022).

At the time of this study, there has been limited research on the role of community pharmacies in the opioid epidemic. While previous research identified corporate culture as a factor in CVS inappropriately dispensing opioids in West Virginia, our research expands significantly on this work by detailing specific store practices, management pressures, and corporate agreements between Walgreens and Purdue (Gonick, 2022). These findings suggest potential reforms to organizational practices that could reduce inappropriate dispensing in the future. For example, state boards of pharmacy might consider limiting the discretion of store managers to override dispensing denials by pharmacists, restricting the share of controlled substances prescriptions that can be dispensed without a prescriber DEA number, imposing requirements that pharmacies eliminate all controlled substances from incentive programs and store metrics, or requiring that CE credits be provided by independent sources.

Our study has both strengths and limitations. Inherently, observational research can only identify associations. These findings do not imply that Walgreens singlehandedly contributed to the opioid epidemic, as other factors (e.g., legislation, regulation, prescriber behavior, and opioid manufacturer practices) likely influenced these outcomes. The OIDA contains multiple documents that did not provide clear context and could be left open to interpretation, as well as documents that retracted key information on the grounds that it referred to confidential business practices. The documents in the archives included only those identified as relevant to litigation, related to a single company, and as a result are likely to represent an incomplete and non-representative sample of all documents produced. Future research could address some of these limitations by considering the opioid dispensing practices of other retail chains and of independent pharmacies. Nonetheless, this information would not be discoverable from other sources without introducing recall bias and social desirability bias; documents released in discovery include details that might otherwise have been redacted or reframed by companies in order to protect their public image.

### 4.1 Conclusions

Multiple retail chain pharmacies have been held legally liable for dispensing practices that may have contributed to the opioid epidemic. Our results identified specific practices at Walgreens stores and distribution centers, as well as at the corporate level, associated with improper opioid dispensing. Factors that influenced dispensing included store-level procedures, management pressure, distribution center activities, and pharmaceutical company sponsorship. These findings provide information that could help regulate future opioid dispensing, as well as suggesting guidelines that could prevent inappropriate dispensing of other medications that have potential for addiction and reduce future overdoses. Although our study focused on Walgreens, retail pharmacies under other ownership may also have engaged in similar practices. Overall, these findings suggest ways to modify organizational policies and procedures in pharmacies that could help prevent future drug epidemics.

## Funding

This research received no specific grant from any funding agency in the public, commercial or not-for-profit sectors.

## Ethical approval and protection of human participants

The research was conducted using an existing dataset that can be accessed freely by the public without special permission or application; as a result, the information is not defined as ‘‘private’’ and was excluded from institutional review board assessment on the grounds that it did not involve human subjects.

## Data availability

The data supporting the conclusions of this article are publicly available at the University of California Industry Documents Library: https://www.industrydocuments.ucsf.edu/

## Contributors

All authors have approved the final article.

## CRediT authorship contribution statement

**Clever Chiu:** Conceptualization, Methodology, Validation, Investigation, Data curation, Writing – original draft, Visualization. **Anthony Wong:** Conceptualization, Methodology, Validation, Investigation, Data curation, Writing – original draft, Visualization. **James Chhen:** Conceptualization, Methodology, Validation, Investigation, Data curation, Writing – original draft, Visualization. **Alfred-John (A.J.) Roderos:** Conceptualization, Methodology, Validation, Investigation, Data curation, Writing – original draft, Visualization. **Dorie E. Apollonio:** Conceptualization, Methodology, Writing – review & editing, Supervision, Project administration.

## Declaration of Competing Interest

The authors declare that they have no actual or potential competing financial interests.

## Appendix Supplementary materials

Image, application 1

## References

[bib0001] Abbasi, K. and Smith, R.. 2003. “No more free lunches.” May 31, 2003. https://www.ncbi.nlm.nih.gov/pmc/articles/PMC1126035/. 10.1136/bmj.326.7400.1155 PMC1126035 12775587

[bib0002] Alnsour, M. 2022. “Using modified grounded theory for conducting systematic research study on sustainable project management field.” November 1, 2022. https://doi.org/10.1016/j.mex.2022.101897. 10.1016/j.mex.2022.101897 PMC9646964 36385919

[bib0003] Bandyopadhyay S. (2019). An 8,000-year history of use and abuse of opium and opioids: how that matters for a successful control of the epidemic ? (P4.9-055). Neurology.

[bib0004] Bedene A., Dahan A., Rosendaal F.R., van Dorp EL.A. (2022). Opioid epidemic: lessons learned and updated recommendations for misuse involving prescription versus non-prescription opioids. Expert Rev. Clin. Pharmacol..

[bib0005] Bero L. (2003). Implications of the tobacco industry documents for public health and policy. Annu. Rev. Public Health.

[bib0006] Bratton, E. 2013. “Drug enforcement administration (DEA) agreement action items, version 2.” June 26, 2013. https://www.industrydocuments.ucsf.edu/docs/#id=jkph0257.

[bib0007] Caleb Alexander G., Mix LA., Choudhury S., Taketa R., Tomori C., Mooghali M., Fan A. (2022). The opioid industry documents archive: a living digital repository. Am. J. Public Health.

[bib0008] Carriedo A., Pinsky I., Crosbie E., Ruskin G., Mialon M. (2022). The corporate capture of the nutrition profession in the USA: the case of the academy of nutrition and dietetics. Public Health Nutr..

[bib0009] CDC (Centers for Disease Control and Prevention). 2022a. “Overview | drug overdose | CDC injury center.” May 18, 2022. https://www.cdc.gov/drugoverdose/deaths/prescription/overview.html.

[bib0010] CDC (Centers for Disease Control and Prevention). 2022b. “U.S. opioid dispensing rate maps | Drug overdose | CDC injury center.” October 19, 2022. https://www.cdc.gov/drugoverdose/rxrate-maps/index.html.

[bib0011] CDC (Centers for Disease Control and Prevention). 2023. “Opioid data analysis and resources | Opioids | CDC.” April 5, 2023. https://www.cdc.gov/opioids/data/analysis-resources.html.

[bib0012] Chudnow, J.. 2010. “16 letters from the oviedo police department to 2 walgreens pharmacists and 2 Walgreen Co. executives.” November 23, 2010. https://www.industrydocuments.ucsf.edu/docs/#id=mmmh0257.

[bib0013] Cicero TJ., Inciardi JA., Muñoz A. (2005). Trends in abuse of OxyContin® and other opioid analgesics in the United States: 2002-2004. J. Pain.

[bib0014] Coffin, P., Long, K., and McMahan, V.. 2022. “Substance use trends in san francisco | Center on substance use and health.” CSUH SF. October 6, 2022. https://www.csuhsf.org/substance-use-trends-san-francisco.

[bib0015] Denning, D. 2000. “Re : walgreens࿽ request for money.” January 17, 2000. https://www.industrydocuments.ucsf.edu/docs/#id=thmh0257.

[bib0016] Gale AH. (2016). Drug company compensated physicians role in causing america's deadly opioid epidemic: when will we learn?. Mo. Med..

[bib0017] Gasdia, R. 1997. “*Re*: walgreens.” February 9, 1997. https://www.industrydocuments.ucsf.edu/docs/#id=stbx0257.

[bib0018] Gonick SA. (2022). Opioid litigation: lessons learned from a retail pharmacy settlement. Am. J. Law Med..

[bib0019] Gubbins, T. 2010. “Oxycodone sales.” August 2, 2010. https://www.industrydocuments.ucsf.edu/docs/#id=qqmh0257.

[bib0020] Gunnarsson J.A., Ruskin G., Stuckler D., Steele S. (2023). Big food and drink sponsorship of conferences and speakers: a case study of one multinational company's influence over knowledge dissemination and professional engagement. Public Health Nutr..

[bib0021] Jaeger, R. 2018. “Incident at Store 7832.” February 16, 2018. https://www.industrydocuments.ucsf.edu/docs/#id=pgcx0257.

[bib0022] Leung PT.M., Macdonald EM., Stanbrook MB., Dhalla IA., Juurlink DN. (2017). A 1980 letter on the risk of opioid addiction. N. Engl. J. Med..

[bib0023] Mail, R.X. 2012. “Materials for today's controlled substance action plan videoconference.” June, 11, 2012. https://www.industrydocuments.ucsf.edu/docs/#id=mfph0257.

[bib0024] Martin, BA. 2011. “*Re*: high Quantity Stores 682971.” January 11, 2011. https://www.industrydocuments.ucsf.edu/docs/#id=mfnh0257.

[bib0025] Meier B. (2007). https://www.nytimes.com/2007/05/10/business/11drug-web.html.

[bib0026] OIDA (Opioid Industry Documents Archive). 2023 “Opioid industry documents.” Accessed May 18, 2023. https://www.industrydocuments.ucsf.edu/opioids.

[bib0027] Porter J., Jick H. (1980). Addiction rare in patients treated with narcotics. N. Engl. J. Med..

[bib0028] Raval, S. 2010. “Re: question about Fla board holding Rph's liable for excessive C2 Ax's.” July 6, 2010. https://www.industrydocuments.ucsf.edu/docs/#id=symh0257.

[bib0029] Rosenblum A., Marsch LA., Joseph H., Portenoy RK. (2008). Opioids and the treatment of chronic pain: controversies, current status, and future directions. Exp. Clin. Psychopharmacol..

[bib0030] Scifo, T. 2001. “RE: walgreen's Annual Wisconsin Meeting (Addendum).” February 2, 2001. https://www.industrydocuments.ucsf.edu/docs/#id=qfcx0257.

[bib0031] Seid, S. 2001. “FW: walgreen.” October, 19, 2001. https://www.industrydocuments.ucsf.edu/docs/kxmh0257.

[bib0032] Sposato, C. 2001. “RE: walgreen Florida Speaker Program.” September 17, 2001. https://www.industrydocuments.ucsf.edu/docs/#id=ggcx0257.

[bib0033] Steele S., Sarcevic L., Ruskin G., Stuckler D. (2022). Confronting potential food industry ‘front groups’: case study of the international food information council's nutrition communications using the UCSF food industry documents archive. Glob. Health.

[bib0034] “Summary of Walgreens Dispensing Data Quality, from 2005 to 2020 : For Walgreens Stores in San Francisco, Walgreens Stores Outside San Francisco, and 12 Selected Walgreens Stores in San Francisco.” 2020. 2020. https://www.industrydocuments.ucsf.edu/docs/#id=zhwx0257.

[bib0035] USAO. 2015. “Walgreens agrees to pay a record settlement of $80 million for civil penalties under the controlled substances Act.” March 12, 2015. https://www.justice.gov/usao-sdfl/pr/walgreens-agrees-pay-record-settlement-80-million-civil-penalties-under-controlled.

[bib0036] US DEA (United States Drug Enforcement Agency). 2012a. “Order to show cause, in the matter of Walgreen Co.” September 13, 2012. https://www.industrydocuments.ucsf.edu/docs/#id=pqmh0257.

[bib0037] US DEA (United States Drug Enforcement Agency). 2012b. “Order to show cause, in the matter of Walgreen Co. (d/b/a/Walgreens # 03629), as to why the drug enforcement administration should not revoke walgreens 03629’s DEA certificate of registration BW4713992.” November 26, 2012. https://industrydocuments.ucsf.edu/docs/#id=yhvx0257.

[bib0038] Van Zee A. (2009). The promotion and marketing of oxycontin: commercial triumph, public health tragedy. Am. J. Public Health.

[bib0039] Walgreen Co. 2010. “Pharmacy manager bonus program.” December 2010. https://www.industrydocuments.ucsf.edu/docs/#id=hqmh0257.

[bib0040] “Walgreens Code of Business Conduct Lead: IC10001237824.” 2014. August 28, 2014. https://www.industrydocuments.ucsf.edu/docs/#id=rymh0257.

[bib0041] “Walgreens Distribution and Dispensing of Nine Prescription Opioids in Dosage Units by Walgreens Pharmacy, by Year : For 2006-2020, in San Francisco, California.” 2020. 2020. https://www.industrydocuments.ucsf.edu/docs/#id=ghwx0257.

[bib0042] Walgreens (Firm). 2008. “Walgreens internal audit report : drug enforcement administration (DEA) compliance - perrysburg distribution center.” December 22, 2008. https://www.industrydocuments.ucsf.edu/docs/#id=sfnh0257.

[bib0043] Walgreens (Firm), Apis (Firm). 2013a. “Hotline incident- compliance view: CM-HTL-IN-118340970: ethics and compliance employee hotline.” July 23, 2013. https://www.industrydocuments.ucsf.edu/docs/#id=xhcx0257.

[bib0044] Walgreens (Firm), Apis (Firm). 2013b. “Hotline incident- compliance view: IC10000316819: ethics and compliance employee hotline.” September 2, 2013. https://www.industrydocuments.ucsf.edu/docs/#id=lhcx0257.

[bib0045] Walgreens (Firm), Apis (Firm). 2015. “Hotline incident- compliance view: IC10005617104: ethics and compliance employee hotline.” October 29, 2015. https://www.industrydocuments.ucsf.edu/docs/#id=nhcx0257.

[bib0046] Walgreens (Firm), Apis (Firm). 2017. “Internal investigation form - compliance view: IC10007443194: investigation form: store #06762.” July 21, 2017. https://www.industrydocuments.ucsf.edu/docs/#id=tgcx0257.

[bib0047] Yakubi H., Gac B., Apollonio DE. (2022). Marketing opioids to veterans and older adults: a content analysis of internal industry documents released from State of Oklahoma v. Purdue Pharma LP, et Al. J. Health Polit. Policy Law.

[bib0048] Zaltz DA., Bisi LE., Ruskin G., Hoe C. (2022). How independent is the international food information council from the food and beverage industry? A content analysis of internal industry documents. Glob. Health.

