# Supplementary material for: Retail chain pharmacy opioid dispensing practices from 1997 to 2020: A content analysis of internal industry documents
Source: Drug Alcohol Depend Rep. 2023 Nov 2;9:100199. doi: 10.1016/j.dadr.2023.100199 (PMC10713840; doi:10.1016/j.dadr.2023.100199)
Supplement: Supplementary file 1 [file mmc1.docx]

## Retail chain pharmacy opioid dispensing practices from 1997 to 2020: A content analysis of internal industry documents

## Appendix

**Table A1. San Francisco Walgreens opioid dispensing data quality, 2005-2020**. San Francisco Walgreens stores were more likely to dispense opioids inappropriately than stores outside San Francisco: 4.13% (versus 3.16% elsewhere) of opioid prescriptions dispensed lacked a prescriber’s DEA number. 15.05% (versus 13.69% elsewhere) of opioid prescriptions dispensed lacked a unique prescriber’s DEA number, and 97.05% (versus 95.5% elsewhere) of opioid prescriptions dispensed lacked a diagnosis code. Source: UCSF Opioid Industry Documents Archive; data compiled by the authors


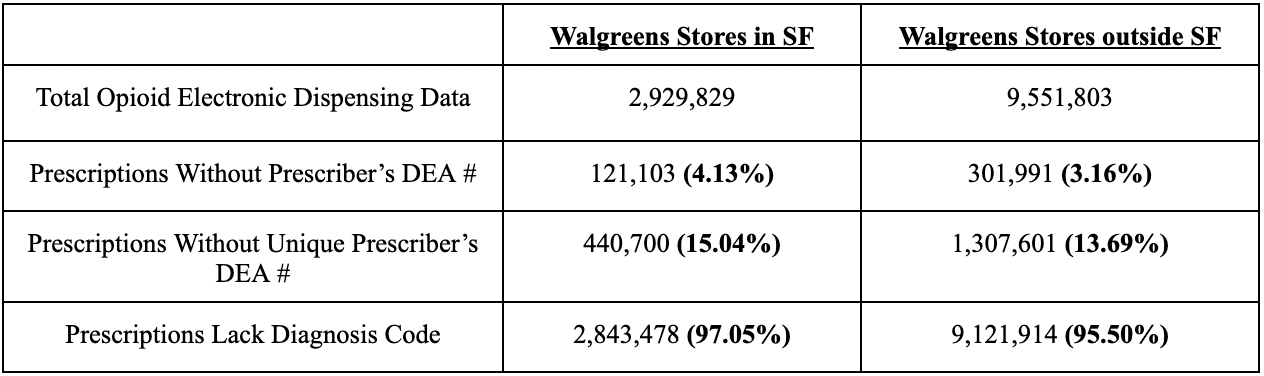


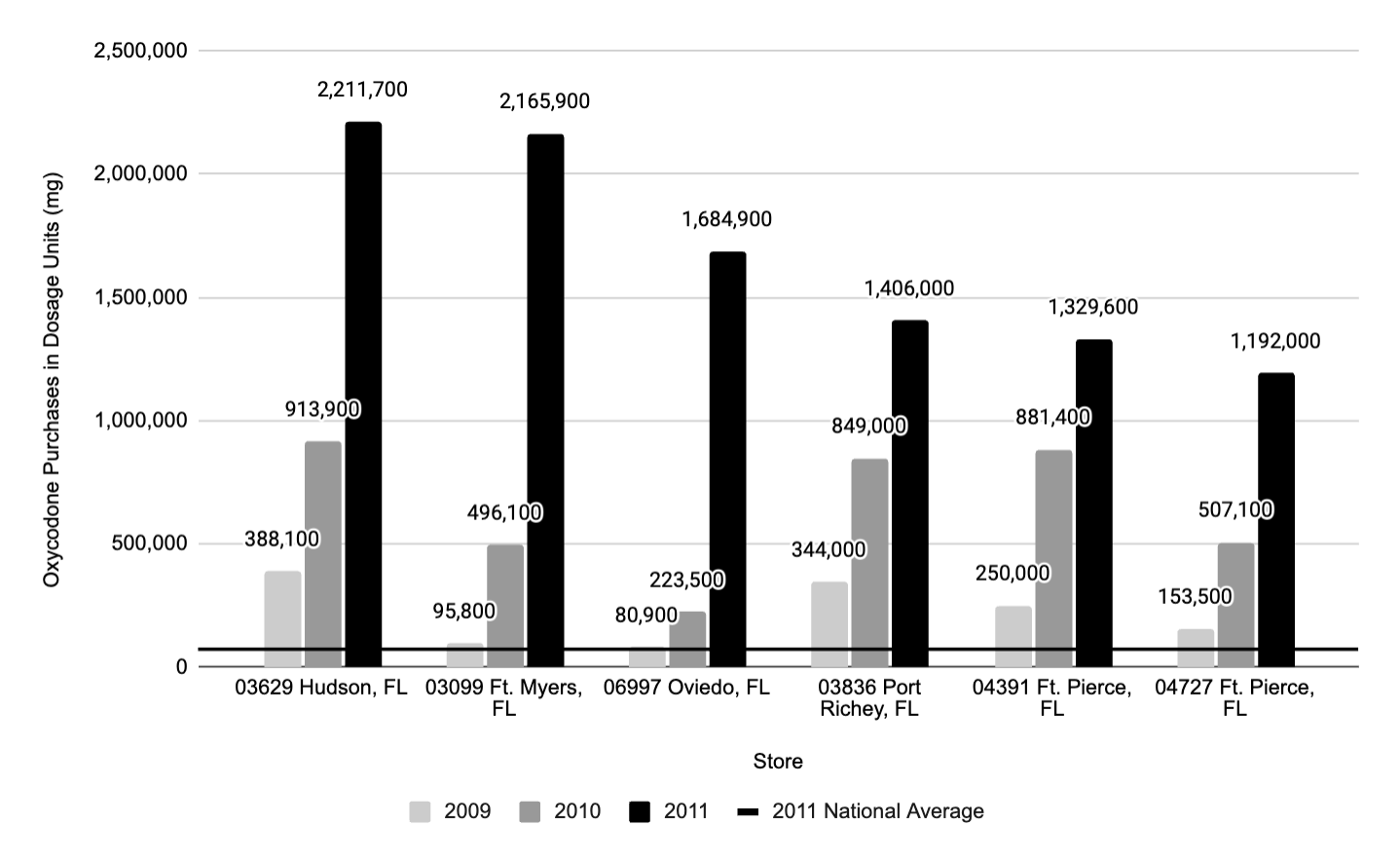


**Figure A1. Oxycodone purchases for six Walgreens retail pharmacies In Florida vs. national average rates.** Purchasing data of oxycodone in dosage units between 2009 to 2011 of six Walgreens pharmacies supplied by the Jupiter distribution center versus 2011 national average of 73,000 dosage units. Source: UCSF Opioid Industry Documents Archive; data compiled by the authors
